# Informing resilience building: FAO’s Surveillance Evaluation Tool (SET) Biothreat Detection Module will help assess national capacities to detect agro-terrorism and agro-crime

**DOI:** 10.1186/s42522-021-00045-8

**Published:** 2021-07-19

**Authors:** Gisela Vasconcelos Gioia, Gaël Lamielle, Ryan Aguanno, Ihab ElMasry, Béatrice Mouillé, Cristian De Battisti, Angélique Angot, Fanny Ewann, Adrien Sivignon, Daniel Donachie, Orr Rozov, Étienne Bonbon, Frédéric Poudevigne, Sophie VonDobschuetz, Ludovic Plée, Wantanee Kalpravidh, Keith Sumption

**Affiliations:** 1 Food and Agriculture Organization of the United Nations (FAO), Rome, Italy; 2 International Criminal Police Organization (INTERPOL), Lyon, France; 3 World Organisation for Animal Health (OIE), Paris, France

**Keywords:** Agro-terrorism, Agro-crime, Surveillance, Biothreat, Needs assessment, Pathogens

## Abstract

Attacks using animal pathogens can have devastating socioeconomic, public health and national security consequences. The livestock sector has some inherent vulnerabilities which put it at risk to the deliberate or accidental spread of disease. The growing concern of countries about the risks of agro-terrorism and agro-crime has led to efforts to prepare against potential attacks. One recent international effort is the launch of a joint OIE, FAO and INTERPOL project in 2019 to build resilience against agro-terrorism and agro-crime targeting animal health with the financial support of the Weapons Threat Reduction Programme of Global Affairs Canada. Given the importance of strong animal health surveillance systems for the early and effective response to agro-terrorism and agro-crime, the project will use the FAO Surveillance Evaluation Tool (SET) and its new Biothreat Detection Module to evaluate beneficiary countries’ capacities to detect criminal or terrorist animal health events. This paper presents the development of the new SET Biothreat Detection Module and how it will be used to evaluate surveillance for agro-terrorism and agro-crime animal disease threats. The module will be piloted in early 2021 and, once finalized, will be used by beneficiary countries of the joint OIE-FAO-INTERPOL project. Results from evaluations using SET and its Biothreat Detection Module are expected to provide a baseline from which countries can build targeted capacity for animal disease surveillance including early detection and investigation of potential terrorist or criminal events involving zoonotic and non-zoonotic animal pathogens.

## Introduction

Before the United Nations Biological Weapons Convention (BWC) came into effect in 1975 [1], the study and use of animal pathogens as biological weapons was not exceptional. Countries such as the United States, former USSR, Canada, Japan, Germany and the United Kingdom pursued bioweapons programs at various points that included research on many animal pathogens which cause diseases such as anthrax, African swine fever (ASF), brucellosis, Foot-and-mouth disease (FMD), glanders, New Castle disease and Rinderpest [2, 3]. Today, most countries have joined the Convention which requires all States Parties to never develop, produce, stockpile, acquire or retain weapons using biological agents or toxins [1]. However, especially after the 2001 anthrax attacks in the United States, concerns have grown regarding the development and use of biological weapons by non-state actors. This led to the development of the United Nations Security Council Resolution 1540 in 2004, which requires all Member States to adopt laws and effective measures to prevent the proliferation of nuclear, chemical or biological weapons and their means of delivery by non-state actors, in particular for terrorist purposes [4].

Although rare, reports exist throughout history of non-state actors using or threatening to use biological agents against animals [2, 3, 5, 6]. These acts, when implemented to coerce politico-social objectives, are classified as agro-terrorism, a sub-set of bioterrorism specifically targeting livestock and crop production [3, 7]. When motivated by financial or personal gain, these deliberate acts are considered as agro-crimes [8]. The serious economic, social, public health and security impacts of a major animal disease outbreak, the inherent vulnerabilities of the livestock sector and of the exotic pet and wildlife trade, and the perceived advantages of biological weapons compared to other weapons makes agro-terrorism appealing to ill-intended non-state actors [3, 9–11]. As potential biological weapons, animal pathogens can be easier to obtain, handle and be released with less expertise and technology compared to other unconventional weapons [9, 10]. Many biological agents are environmentally resistant and are not included in vaccine programs, making them attractive candidates as bioweapons. The acquisition and production costs can also be considerably lower. According to Gyles (2010), producing biological weapons may cost approximately 10 million USD while nuclear weapons require approximately 1 billion USD to develop. In addition, non-zoonotic pathogens would be safer for a terrorist to manipulate but can still cause serious harm to a country’s economy and food security. For instance, ASF could be introduced in a country or farm via contaminated items [12]. Attacks on animals may also be perceived as less ethically and morally discomforting compared to harming humans [6]. On the other hand, 80% of agents with potential bioterrorist use are zoonotic [13].

In terms of vulnerabilities, many factors make animals more vulnerable to bioterrorism compared to humans and crops. Firstly, current intensive farming practices can facilitate and accelerate the spread of diseases [9, 14, 15]. Secondly, insufficient biosecurity or disease surveillance in animals in some countries, facilitate the introduction and favor greater spread of diseases before they are detected and control measures applied [9, 16]. Thirdly, there is usually a limited number of veterinarians, veterinary paraprofessionals (VPP), community animal health workers (CAHWS) and other animal health professionals, especially in the field, capable of detecting exotic or eradicated diseases that have similar clinical signs and symptoms than endemic disease. This may also delay detection and implementation of appropriate control measures [9, 14, 15]. Moreover, many animals will likely be naïve or unvaccinated to exotic or eradicated diseases which would potentially facilitate the spread and severity of the disease [15]. Fourthly, animals’ congregation and movement within and between countries are greater than those of crops, notably through transhumance or for commercial purposes. The movement of animals and animal products have greatly increased in the past years due to trade but also to the globalization of livestock value chains. Today, the different production phases such as reproduction, fattening and slaughter are rarely done at the same location [14, 15]. The increasing and prosperous illegal trade of exotic pets and wildlife has also been pointed out as a major gateway for infectious diseases [11]. Finally, advancements in the areas of molecular biology, genomics, bio-informatics and genetic engineering have opened a wide range of positive and dangerous possibilities such as the development of genetically-modified disease-causing organisms [16].

In view of such risks, several authors and reports have called for the development of international and national capacities for the prevention, detection and response to agro-terrorism and agro-crime [6, 9, 10, 13, 15]. In response, the international community has promoted resilience against agro-terrorism and agro-crime with the development of strategies and activities on biological threat reduction [13, 17–19]. One recent effort is the creation of a consortium between the World Organisation for Animal Health (OIE), the Food and Agriculture Organization of the United Nations (FAO) and the International Criminal Police Organization (INTERPOL) to enhance prevention and response to agro-terrorism and agro-crime affecting animals. The consortium signed a joint project in October 2018 on “Building resilience against agro-terrorism and agro-crime” with the financial support of the Weapons Threat Reduction Programme (WTRP) of Global Affairs Canada [20].

The aim of the project is the sustainable increase of global resilience against animal health emergencies arising from agro-terrorism and agro-crime by improving coordination between the animal health and law enforcement sector. The project focuses on the three priority regions of the Middle East, North Africa, and South East Asia where previous work of the three organizations identified gaps in various aspects of emergency management that may make them vulnerable to emergencies resulting from agro-crime and agro-terrorism [21]. The first phase of the project focuses on evaluating the current capacity of target regions and seeking to find innovative and sustainable solutions to emergency management. Using this evidence, a second phase of trainings, including the development of fit-for-purpose guidance, tools, and workshops, will be implemented followed by a third phase that will use regional simulation exercises and a large international simulation exercise to challenge and test the lessons learned and the efficiency of international cooperation.

Lastly, through a continuous coordination and communication phase, project outputs will be shared and used to produce communications and advocacy material to encourage collaboration between veterinary and law enforcement sectors. The project will culminate with a global conference on animal health and welfare emergency management to review and share lessons learned and knowledge acquired and rally support from the international community for an all-hazards and multi-sectoral approach to emergency management [20].

To evaluate needs and capacities of beneficiary countries, the project will use the OIE Tool for the Evaluation of the Performance of Veterinary Services (PVS) and three FAO tools, namely the Laboratory Mapping Tool (LMT), the Good Emergency Management Practices (GEMP) and the Surveillance Evaluation Tool (SET). Relevant results of past Joint External Evaluations (JEE) lead by the World Health Organization (WHO) and of recent reports regarding biological security needs, trends and priorities by the United Nations Interregional Crime and Justice Research Institute (UNICRI) will also be used when available [22]. Additional questions or modules regarding agro-terrorism and agro-crime prevention, detection or response are currently being developed for the OIE PVS and the three FAO tools (LMT, GEMP and SET). The outcomes of PVS evaluations will provide a wider perspective on the current performance of veterinary services and resilience against agro-terrorism and agro-crime targeting animals, whereas the FAO SET, LMT and GEMP tools will respectively provide a more detailed evaluation of national animal disease surveillance, diagnostic and emergency preparedness capacities [23–26]. The LMT Biothreat Module will be used to evaluate the capacity of veterinary laboratories involved in the handling and testing of samples from potential terrorist or criminal induced animal disease outbreaks. The FAO GEMP will also include an additional module to evaluate capacities and provide training to beneficiary countries on emergency management of agro-terrorism and agro-crime events. Lastly, the new Biothreat Detection Module developed for the FAO SET is the focus of this paper.

Although countries may take several actions to prevent agro-terrorism or agro-crime, emphasis should be placed on early detection to counter such occurrences [27]. Therefore, effective preparedness and response frameworks are needed. This requires well-functioning surveillance systems which allow the early detection and response to an outbreak or event, the tracing of cases for disease containment and perpetrator identification, and the confirmation of the end of an outbreak [27]. In addition, animals may act as sentinels for bioterrorism attacks with zoonotic pathogens [13]. Thus, to inform capacity building efforts and biothreat reduction policies, the joint OIE-FAO-INTERPOL project will evaluate beneficiary countries’ capacities to detect deliberate animal health events using FAO’s Surveillance Evaluation Tool (SET) and its new Biothreat Detection Module along with other tools of the consortium. In this paper, we present the newly developed SET Biothreat Detection Module and how it will evaluate the capacity of countries to conduct effective surveillance for agro-terrorism and agro-crime.

## Main text

### The FAO Surveillance Evaluation Tool (SET)

SET was developed in 2017 by FAO upon request from beneficiary countries in Africa under the Global Health Security Agenda (GHSA). The tool’s objective is to provide countries with detailed guidance and recommendations to improve their national animal disease surveillance systems. SET was adapted from the OASIS tool (“Outil d’Analyse des Systèmes de Surveillance”), developed by the French Agency for Food, Environmental and Occupational Health & Safety (ANSES) [28], and has been used in 18 countries in Africa and Asia to date [24]. Reports of past SET evaluations whose publication were authorized by the respective national Chief Veterinary Officers (CVO) are available at the FAO webpage for SET [24].

**Fig. 1:**
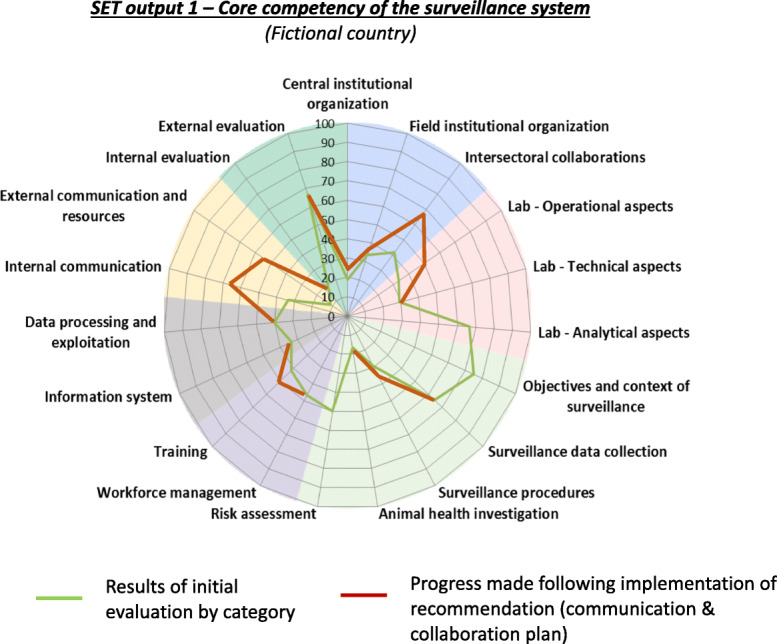
Example of graphical output of the FAO Surveillance Evaluation Tool (SET) using figurative results of a fictive country. The spider graph shows results by SET category of a fictive country who has conducted two SET evaluations. Each of the 19 SET categories form an individual axis whose value varies from 0 to 100%. The green line shows results for the first SET evaluation and the red line shows results for the second SET evaluation conducted 3–5 years after the initial evaluation. The closer either line is positioned to the outer border of the graph, the higher the capacity of the country in the respective category and vice-versa. The graphic shows the progress the country has made after implementing the recommendations of the first SET evaluation, particularly the development and implementation of a communication and inter-sectoral collaboration plan

SET is an Excel-based tool with 90 indicators divided into seven areas and 19 categories specific to animal disease surveillance (Table 1). The evaluated country receives a score from 4 to 1 based on their current capacity in each indicator. An example of a SET indicator is provided in Table 2. Once all 90 indicators are scored, the tool automatically generates graphics depicting the system’s strengths and weaknesses (Fig. 1).

**Table 1:** Overview of SET areas, categories and indicators

Area	Category	Number of indicators	Summary of topics covered by indicators
**Institutional organization**	Central institutional organization	7	Existence of an operational management structure; Existence of steering and technical/scientific committees; Existence of formal description of the system’s organization and operations; Vertical coordination and supervision of field level units; and Adequacy of central level resources.
	Field institutional organization	8	Formalization, coverage and representativeness of field units; Vertical and horizontal coordination between field units; Supervision of field units; and Adequacy of resources.
	Intersectoral collaborations	4	Coordination with private, public health and environmental sectors.
**Laboratory**	Operational aspects	2	Effective integration of laboratories in the surveillance system; and Adequacy of resources.
	Technical aspects	8	Quality assurance mechanisms including tests, laboratory reagent control and inter-laboratory proficiency testing; Work standardization between laboratories; Field laboratory support; and Relevance, sensitivity and specificity of tests.
	Analytical aspects	3	Laboratory data management; and Timeliness and quality of laboratory reports.
**Surveillance activities**	Objectives and context of surveillance	4	Quality and formalization of surveillance objectives; and Relevance of priority diseases
	Surveillance data collection	14	Existence and quality of surveillance plans; Existence and quality of data collection tools; Existence and quality of case definitions; Completeness and timeliness of disease reports; Appropriate sample collection; and Timeliness of results delivery.
	Surveillance procedures	9	Existence and quality of surveillance protocols; Existence and quality of active surveillance activities; Existence of wildlife surveillance; and Existence of vector surveillance.
	Animal health investigations	2	Availability of animal health investigation teams; and Quality and timeliness of investigations.
	Risk assessment	2	Implementation and usefulness of animal health risk assessments.
**Workforce**	Workforce management	5	Planning, Terms of Reference (ToRs) and qualification of epidemiology staff; and Existence of sufficient manpower for surveillance.
	Training	4	Existence and overall quality of initial and refresher surveillance staff trainings; and Adequacy of resources for trainings.
**Data management**	Information system	2	Adequacy and quality of the data management system for the needs of the system.
	Data processing and exploiting	5	Existence of protocols, quality and timeliness of data entry, validation and analysis; Existence of sufficient trained staff for data entry, management and analysis; and Adequacy of resources for data management and analysis.
**Communication**	Internal communication	4	Existence, timeliness and completeness of surveillance reports; Existence and timeliness of feedback to field actors; and Existence of horizontal and vertical communication mechanisms within the surveillance system.
	External communication	3	Existence and timeliness of newsletters; Existence and implementation of an external communication policy; and Adequacy of resources for communication.
**Evaluation**	Internal evaluation	2	Existence, quality, timeliness and use of performance indicators for the continuous improvement of the surveillance system.
	External evaluation	2	Existence and use of external evaluations for improvement of the surveillance system.

**Table 2:** SET indicator number 34

#	Indicator	Score 4	Score 3	Score 2	Score 1
**34**	Level of detail, accuracy, and formalization of objectives	Objectives are well detailed and completely formalized, rendering it possible to estimate disease prevalence or assess the probability of detection in conformance with the nature and purpose of the system.	Objectives could benefit from the addition of some minor details and formalization.	Objectives require significant additional details and formalization.	Objectives are not formalized, detailed, or relevant.

Each indicator in SET is scored from 4 to 1. Score 4 represents full capacity, Score 3 represents moderate capacity, Score 2 represents low capacity and Score 1 represents very low capacity. Above is the indicator 34 of SET under the area of surveillance activities and the category of surveillance objectives

Scoring is based on information obtained from interviews with stakeholders from central, sub-national and field levels of the surveillance system as well as a thorough review of relevant documentation (e.g. surveillance plans, protocols, legislation, etc.). This is done during the first week of a 12-day mission in the target country. Stakeholders vary among countries but generally include the veterinary service’s epidemiology unit, field veterinary officers, veterinary laboratories, VPPs and CAHWS, multi-sectoral partners (e.g. Ministry of Health, Ministry of Environment/Wildlife, One Health platforms), the private sector, border inspection posts, slaughterhouses, markets and more. These stakeholders are identified in close collaboration with veterinary services focal points during the preparatory phase of the mission, which usually begins 1 month before the mission itself. It is also during the preparatory phase that the evaluation teams, which include SET experts and focal points from national veterinary services, are formed.

**Fig. 2:**
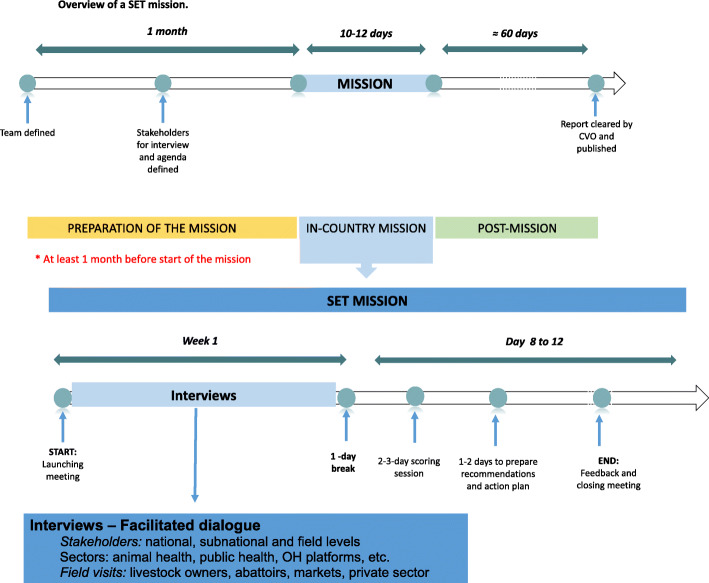
Overview of a SET mission. After at least 1 month of preparation, the in-country SET mission is conducted for 10–12 days. After a launching meeting with key decision-makers, the first week of the mission is dedicated to interviews with all relevant stakeholders. The second week of the mission is dedicated to scoring all 90 indicators of SET and 32 indicators of the SET Biothreat Detection Module (if used), and the development of recommendations and a first draft of the action plan for improvement of the country’s animal disease surveillance system. The second week ends with a closing meeting presenting the main results and recommendations to key decision-makers. The SET report is drafted, finalized and cleared for publication by the country’s Chief veterinary officer approximately 60 days after the in-country mission

During the second week of the mission, evaluators score all 90 indicators, analyze the graphical outputs, and conduct an in-depth analysis of the strengths, weaknesses, opportunities and threats (SWOT) of the country’s animal disease surveillance system. The SWOT analysis results are then used to guide the development of recommendations that are specific, measurable, achievable, locally relevant and have realistic timelines. At the end of the mission, the results and recommendations are presented to decision-makers for feedback and a report is drafted. The report, which includes a detailed action plan to improve the country’s animal disease surveillance system, is usually finalized around 60 days after the mission and posted online once cleared by the country’s Chief Veterinary Officer (CVO) [24] (Fig. 2).

### The SET Biothreat Detection Module

Given the importance of strong surveillance systems in the preparedness and response to agro-terrorism and agro-crime, the OIE-FAO-INTERPOL consortium decided to use SET to obtain a detailed understanding of the beneficiary countries’ animal disease surveillance systems. However, the surveillance of potential deliberate animal disease outbreaks, particularly the investigation of these events, require additional coordination and activities beyond that of routine surveillance, such as forensic investigation, forensic sampling and testing, proper chain-of-custody processing, among others [7]. Therefore, a specific Biothreat Detection Module was developed to be used within SET to assess the capacity of countries to detect unusual animal health events that are indicative of agro-terrorism or agro-crime. The Biothreat Detection Module consists of a list of indicators that can be used by interested countries in addition to the core SET indicators during an evaluation mission.

The Module was developed by animal health and law enforcement experts from FAO, OIE and INTERPOL with a background in epidemiological surveillance, animal health emergencies, veterinary diagnostics and biothreat reduction. An initial draft was developed between March and May 2020 based on an extensive literature review of more than 50 documents including international and national strategies and guidelines, workshop reports, peer-reviewed articles, legislation, international conventions and more. The draft was then reviewed by 14 biothreat reduction experts with different technical and geographic backgrounds (Table 3) between July and September 2020. The module is currently expected to be piloted and finalized in early 2021.

**Table 3:** List of external reviewers of the SET Biothreat Detection Module and their technical and geographic background

Background	Technical									Geographic							Gender
Reviewer	Biothreat reduction	Surveillance	Investigation	Emergency	Laboratory	Wildlife	Public health	Law enforcement	Military	North Africa	Middle East	Southeast Asia	Sub-Saharan Africa	Europe	North America	Latin America	
Alessandro Ripani	X									X							Male
Armin Elbers	X	X												X			Male
Fee Zimmermann	X				X	X							X				Female
Gary Flory	X			X											X		Male
Gary A. Vroegindewey	X			X										X			Male
Jacqueline Roberta Soares Salgado	X								X							X	Female
Julie R. Sinclair	X				X												Female
Júlio Gouveia-Carvalho	X		X						X					X			Male
Mirza Qakhon Hatoqay	X							X			X						Male
Rickard Knutsson	X	X			X									X			Male
Sean Shadomy	X	X	X				X								X		Male
Stephen Papagiotas	X		X												X		Male
Zalini Yunis	X							X	X			X					Female

The module includes 32 indicators divided into 7 categories related to the surveillance of potential deliberate animal disease outbreaks, including their investigation (Table 4). Similar to SET, the indicators are scored from 1 to 4 based on the country’s capacity. Scoring will be based on interviews of relevant stakeholders and on the review of relevant documents related to the surveillance of agro-terrorism and agro-crime against animal health. These stakeholders may include law enforcement authorities, customs, military forces, bioterrorism or agro-terrorism focal points in veterinary services and other inter-sectoral partners, laboratories handling and testing samples from suspected deliberate animal health events, among others. Additional relevant documents may include legislation and strategies to counter agro-terrorism or agro-crime, joint criminal and epidemiologic investigation guidelines, memoranda of understanding between veterinary services and law enforcement, lists of pathogens and toxins of concern for agro-terrorism and agro-crime, among others.

**Table 4:** Overview of SET Biothreat Detection Module categories and indicators

Category	Number of indicators	Summary of topics covered by indicators
**Institutional organization**	5	Existence of agro-terrorism and agro-crime committee; Existence of formal documents on the organization and operation of agro-terrorism and agro-crime surveillance; Existence of focal points and mechanisms for national and international collaboration between animal health, law enforcement and other relevant sectors; and Adequacy of resources for surveillance of agro-terrorism and agro-crime.
**Laboratory**	6	Mechanisms in place to meet laboratory epidemiologic and forensic needs; Existence of guidelines for sampling in joint epidemiologic and criminal investigations; Adequacy of resources and existence of laboratory information management system (LIMS) in laboratories involved in joint investigations; and Capacity of the country to differ endemic, foreign, emerging and potentially manipulated pathogens.
**Surveillance activities**	11	Existence and quality of a list of pathogens of concern for agro-terrorism and agro-crime; Knowledge of epidemiological situation of pathogens of concern; Existence of awareness campaigns on exotic and eradicated diseases; Existence and implementation of triggers and mechanisms for secure information sharing between veterinary services and law enforcement; Cross-border animal disease surveillance capacity; and Existence and quality of guidelines on joint epidemiological and criminal investigations.
**Risk assessment**	2	Implementation, quality and use of threat/risk assessments to guide agro-terrorism and agro-crime surveillance activities.
**Workforce**	5	Existence of staff planning, roster of investigators, background checks and trainings on detection, reporting and joint epidemiological and criminal investigations of potential agro-terrorism or agro-crime animal health events.
**Data management**	1	Existence and implementation of mechanisms to secure surveillance data and sensitive information from theft, loss or misuse.
**Evaluation**	2	Implementation and use of joint simulation exercises and after action reviews for improvement of agro-terrorism and agro-crime surveillance.

**Fig. 3:**
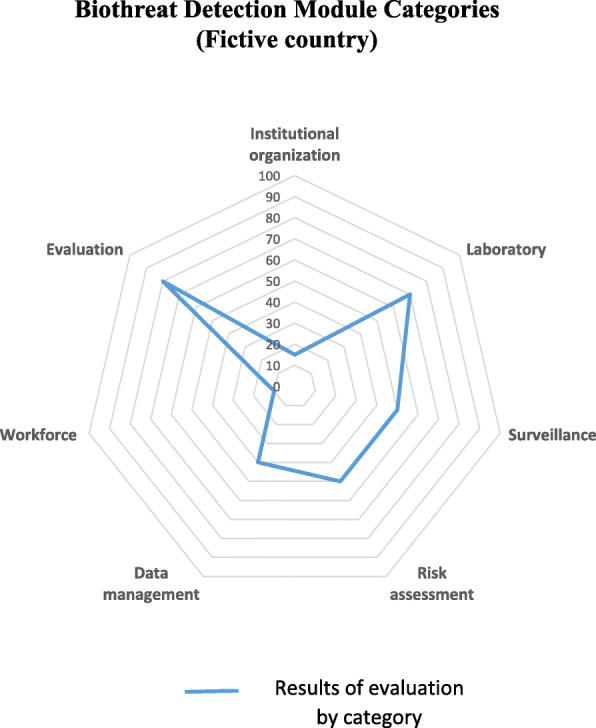
Example of graphical output of the FAO SET Biothreat Detection Module using figurative results of a fictive country. The spider graph shows results by SET Biothreat Detection Module category of a fictive country. Each of the 7 SET Biothreat Detection Module categories form an individual axis whose value varies from 0 to 100%. The blue line shows the results for the SET Biothreat Detection Module from 0 to 100%. The closer the line is positioned to the outer border of the graph, the higher the capacity of the country in the respective category and vice-versa

Once all 32 indicators are scored, the module will automatically generate a spider graph of the country’s strengths and weaknesses in the detection of biological threats (Fig. 3). This graphical output may then be used to develop recommendations to improve national surveillance of agro-terrorism and agro-crime events. Similar to SET, recommendations will be developed using the SMART approach (Specific, Measurable, Achievable, Relevant and Time-bound). For this, the recommendations will be prioritized into short, medium and long-term and detailed in an action plan for improvement of animal health agro-terrorism and agro-crime surveillance.

The 7 categories of the SET Biothreat Detection Module are Institutional Organization, Laboratory, Surveillance, Risk Assessment, Workforce, Data Management and Evaluation. The following sections describe the requirements for effective surveillance of suspected deliberate animal disease outbreaks and how national capacities to implement these activities are evaluated under each category of the SET Biothreat Detection Module.

### Institutional organization category of the SET Biothreat Detection Module

The timely exchange of information between animal health and law enforcement agencies is critical to contain the spread of disease and apprehend the perpetrators in a deliberate outbreak. However, several factors may delay the exchange of information including hesitancy to share information due to its sensitivity, legal barriers, and lack of awareness and guidance on how to collaborate. Lack of clear guidance on: leadership for response activities when a terrorist or criminal event targeting animals is suspected or confirmed, the role and responsibilities of each entity involved, the appropriate communication lines to be followed as well as outreach to the public may cause an overlap of efforts, waste of resources, and delayed detection of the outbreak source and of identification of the perpetrators leading to an overall inefficient response [15, 29, 30].

Thus, the institutional organization category of the SET Biothreat Detection Module evaluates the factors that would create an enabling environment for intersectoral collaboration and efficient detection of potential deliberate animal disease outbreaks. This includes the existence of committees to discuss, develop and review strategies and plans related to agro-terrorism and agro-crime surveillance based on national needs and threats. It also includes the existence of a formal framework that outlines the organization and operation of surveillance activities for the detection of potential deliberate animal health events, the existence of focal points and mechanisms for national and international inter-sectoral collaboration particularly between animal health and law enforcement agencies, and the existence of sufficient resources.

### Laboratory category of the SET Biothreat Detection Module

Initially, veterinary services and law enforcement have the common aim of identifying the causative agent and source of a potentially deliberate outbreak using similar methods. However, the end goal of law enforcement is to determine who committed the criminal offence and bring them to prosecution. To this end, law enforcement agencies use biological and other conventional evidence to build a case for attribution to a specific source and identify perpetrators, partners in crime and victims. Exploitation of criminal evidence may require forensic methods that delve deeper into the characterization of the pathogen and specific processing methods. These methods must be standardized, validated and meet standards for its results to be accepted as evidence in court [31]. Yet, not all forensic laboratories have the necessary equipment for processing and testing animal pathogens and biological toxins while not all veterinary or public health laboratories can perform the necessary forensic analysis of conventional evidence (fingerprints, DNA, etc.). Therefore, mechanisms need to be in place to meet the needs of both the animal health and law enforcement sectors in a joint investigation [30, 32]. This may be done by establishing a network of laboratories across the animal health, law enforcement and other sectors, such as public health laboratories, that have the necessary expertise and meet the required standards to conduct the appropriate diagnostic and forensic analysis. These networks were established along the XXI century in a few countries [15, 30, 33]. Nevertheless, countries should at a minimum be aware of national and international laboratory capabilities across animal health, public health and forensic sectors. Based on this, national plans can be developed, and collaboration agreements established. It is worth noting that these plans should also include mechanisms for increasing laboratory capacity to meet surge demands in case of a nationwide emergency [32].

Moreover, for laboratory results to be accepted as evidence in court, laboratories and field agents must comply with specific chain of custody procedures [31]. These practices ensure the integrity of evidence, demonstrate that evidence has been handled properly at all times and that no misconduct or tampering took place, and include a chronological documentation [32]. Thus, chain of custody must be maintained and documented from the collection, packaging, handling, and transport of samples, to their arrival, analysis, storage and disposal, to the drafting and reporting of laboratory results in court [31–33].

The existence of national mechanisms to meet epidemiologic and forensic laboratory needs during a potentially deliberate animal disease outbreak and the availability of guidelines on proper sample collection, transport, handling and preservation for diagnostic and criminal investigation purposes are evaluated in the laboratory category of the SET Biothreat Detection Module. The module also addresses the availability of sufficient resources in relevant laboratories and the percentage of laboratories with information systems. These information systems are important for evidence tracing, chain of custody maintenance and for timely sharing of laboratory results [33]. Finally, the laboratory category verifies the capacity of the country to differentiate foreign, emerging or manipulated pathogens from those that are already circulating in the country. This would require knowledge on the epidemiological situation of animal diseases in the country as well as access to technologies and expertise for pathogen characterization such as genetic sequencing [14, 33]. While SET and its Biothreat Detection Module focus on the general capacity of laboratory networks, once developed the LMT Biothreat Module will provide valuable details on the specific capacities of veterinary laboratories which can complement SET results with a more complete picture of national diagnostic capacities.

### Surveillance category of the SET Biothreat Detection Module

Any animal disease surveillance system relies on farmers, private veterinarians, VPP, laboratories, CAHWs and other data sources to report disease events. The additional challenge for the detection of deliberate animal health events is that many classic bioterrorism or agro-terrorism pathogens are rare, non-endemic or eradicated. Therefore, the detection should also rely on the capacity of animal health professionals to recognize eradicated or exotic diseases of high risk for agro-terrorism or agro-crime [9, 14, 15, 30]. In light of this, the SET Biothreat Detection Module includes an indicator verifying the existence of awareness building programs to inform these data sources on relevant exotic and eradicated diseases based on the country’s risk situation.

Early response to deliberate outbreaks also requires law enforcement and animal health authorities to share information and collaborate. Sharing of information even before confirming suspicions of a deliberate outbreak can be crucial in the identification of the source, control of the spread of disease and apprehension of the perpetrator. However, information sharing and collaboration between sectors for all outbreaks would be overly burdensome and unnecessary [15, 29, 30]. To address this challenge, a few countries and authors have proposed the development of a list of triggers that should prompt animal health and law enforcement authorities to collaborate [29, 30, 32, 34, 35]. Among the possible triggers suggested by authors is the detection of a disease with an unusual geographic, host or seasonal distribution or with an unusual presentation. For this to be noted, animal health authorities must be aware of the epidemiologic distribution of the diseases circulating in the country and the region. This includes information on the temporal, geographic and host distribution of pathogens and toxins as well as their characteristics such as antigenic variants and genetic sequencing [29, 30, 32]. However, countries may not have enough resources to regularly collect data on all animal pathogens and toxins. A commonly used method to prioritize efforts is to focus on a list of pathogens and toxins of concern for agro-terrorism and agro-crime based on the country’s context and threat landscape that is regularly reviewed and updated. Examples include the List of Select Agents and Toxins of the United States Department of Agriculture (USDA) and the Australia Group Common Control List of Human and Animal Pathogens and Toxins [36, 37]. The existence of such a list of pathogens and toxins of concern, the country’s knowledge on the epidemiological situation of these pathogens and toxins of concern, and the existence of a list of triggers for information sharing are also evaluated under the surveillance category of the SET Biothreat Detection Module.

The module also verifies the existence of guidelines or protocols for information sharing between law enforcement and veterinary services. This is important to allow for the safe exchange of information and to overcome potential barriers to the exchange such as legal restrictions [32]. Moreover, when triggers are met, it is advisable for law enforcement and animal health authorities to quickly verify the suspicion in order to avoid unnecessary use of resources in response to a hoax or false alarm [29, 30]. The module also assesses the existence and implementation of protocols to verify the credibility of a possible agro-crime or agro-terrorism threat as well as the reporting timeliness between sectors of unusual events.

Moreover, disease surveillance at entry points of a country, such as airports, ports, border posts, bus stations and postal services, are crucial for preventing the accidental or deliberate entry of diseases in a country [13]. Therefore, the surveillance category of the SET Biothreat Detection Module evaluates the existence of strategies and plans for cross-border surveillance and the capacity of points of entry to conduct surveillance.

Once a suspected deliberate animal disease outbreak is considered a credible threat, it is usually advisable to conduct a joint epidemiologic and criminal investigation between animal health and law enforcement authorities [29, 30, 32]. Depending on the outbreak, other sectors may be included such as public health authorities when the outbreak involves a zoonotic disease or environmental authorities when it involves wildlife. Although it is recommended for authorities to agree on actions on a case-by-case basis, general guidelines can provide an initial understanding on how to proceed including where, how and who to include in the investigation, how to conduct interviews, how to communicate with farmers, the public and all those along the relevant livestock value chain, and key principles in outbreak investigations and criminal investigations (e.g. sample collection, biosafety and biosecurity procedures, chain of custody procedures, etc.) [29, 30, 32]. Therefore, the existence of joint criminal and epidemiologic investigation guidelines for animal health events is also assessed by the module.

### Risk assessment category

In this category, the module verifies whether the country regularly conducts joint risk assessments for potential agro-terrorism and agro-crime threats involving all relevant stakeholders. The module also verifies whether all the minimum components of a risk assessment are done, and if the results are used in risk management to inform strategies, plans, lists of pathogens and toxins of concern and other relevant documents in the detection of agro-terrorism and agro-crime. These assessments are important in prioritizing efforts in the detection of agro-terrorism and agro-crime [32].

### Workforce category of the SET Biothreat Detection Module

One of the factors affecting the surveillance of agro-terrorism and agro-crime is the availability of staff with the necessary training and expertise [27, 32]. In the workforce category, the SET Biothreat Detection Module verifies the existence of staff planning and of a roster of investigators for the surveillance and investigation of suspected or confirmed deliberate outbreaks. The module also evaluates the existence of trainings for relevant staff of the animal health, law enforcement and other relevant entities on the detection, reporting and investigation of potential or confirmed deliberate animal disease outbreaks. This category also addresses the existence of mechanisms to avoid information concealment, the sharing of false information or the misuse of information by animal health, law enforcement or other staff.

### Data management category of the SET Biothreat Detection Module

The loss, theft or misuse of information could jeopardize criminal investigations and place the country at risk. Ill-intended actors could use the information for several nefarious ends [29, 30, 32]. As such, the category of data management was created to include an indicator to assess the existence of mechanism to prevent the theft, loss or misuse of surveillance data and information.

### Evaluation category of the SET Biothreat Detection Module

In general, simulation exercises are done to test, evaluate, and refine plans and protocols. Joint simulation exercises and trainings between animal health, law enforcement and other relevant entities on important surveillance and joint investigation plans and protocols also build relationships between the sectors and allow staff to gain familiarity and expertise with the principles and methods of the detection of deliberate animal disease outbreaks [29, 30, 32]. The evaluation category of the SET Biothreat Detection Module assesses whether countries regularly conduct simulation exercises with all relevant staff of law enforcement, animal health and other pertinent sectors as well as after action reviews (AAR) following an actual event or exercises. The module also verifies if these joint exercises and AARs lead to corrective measures to improve the detection of agro-terrorism and agro-crime.

## Conclusion

Recent epidemics, especially the current COVID-19 pandemic, show us how their impacts may span far beyond the physical health of people and animals by also affecting livelihoods, economies and food security. The UN Secretary General’s remarks to the Security Council on the COVID-19 pandemic notes that the pandemic also poses serious threats to public security and peace by eroding trust in public institutions, creating stressors that can lead to conflicts in fragile societies, postponing democratic elections or referenda, creating uncertainties that may escalate violence, hindering international, regional and national conflict resolution efforts, and by creating a potential window of opportunity for terrorists while the attention of governments is focused on the pandemic [38]. Yet, despite the potentially devastating impacts of accidental or deliberate events using animal pathogens, surveys to date and the current pandemic have shown that most countries are not prepared for such threats [2, 7, 22]. This is also mentioned by the UN Secretary General who stated “the weaknesses and lack of preparedness exposed by this [COVID-19] pandemic provide a window onto how a bioterrorist attack might unfold – and may increase its risks. Non-state groups could gain access to virulent strains that could pose similar devastation to societies around the globe [38].”

The SET Biothreat Detection Module was initially developed to evaluate national agro-terrorism and agro-crime surveillance capacities and needs to inform subsequent capacity development efforts in beneficiary countries under the joint FAO, OIE and INTERPOL “Building resilience against agro-crime and agro-terrorism” project. Once piloted and finalized, the module could also be used with SET by other interested countries to obtain a baseline of country capacity for animal disease surveillance including early detection and investigation of potential terrorist or criminal events involving zoonotic and non-zoonotic animal diseases. FAO is currently planning the organization of trainings on the use of SET and its Biothreat Detection Module precisely to expand the network of evaluators throughout the world that may lead national missions.

The end result of SET missions is an action plan with detailed short, medium and long-term recommendations to improve national animal disease surveillance systems. The SET Biothreat Detection Module will have the same final output to guide governments on how to improve the early detection of potential agro-terrorism and agro-crime events. Moreover, evaluations using SET and its new Biothreat Detection Module can be repeated every 3–5 years or upon request from countries to show progress following implementation of recommendations. The expectation is that such evaluations will thus support ongoing and future efforts to build resilience against agro-terrorism and agro-crime worldwide.

## Data Availability

Not applicable.
